# Dysfunction of striatal MeCP2 is associated with cognitive decline in a mouse model of Alzheimer's disease

**DOI:** 10.7150/thno.68439

**Published:** 2022-01-01

**Authors:** Sangjoon Lee, Tae Kyoo Kim, Ji Eun Choi, Yunjung Choi, Minsu You, Jeewon Ryu, Yoo Lim Chun, Suji Ham, Seung Jae Hyeon, Hoon Ryu, Hye-Sun Kim, Heh-In Im

**Affiliations:** 1 Convergence Research Center for Diagnosis, Treatment and Care System of Dementia, Korea Institute of Science and Technology (KIST), Seoul 02792, Korea.; 2 Department of Pharmacology and Biomedical Sciences, Seoul National University College of Medicine, Seoul 03080, Korea.; 3 Brain Science Institute, Korea Institute of Science and Technology (KIST), Seoul 02792, Korea.; 4 Department of Biochemistry, Hanyang University College of Medicine, Seoul 04763, Korea.; 5 Seoul National University College of Medicine, Bundang Hospital, Sungnam 13620, Korea.; 6 Division of Bio-Medical Science & Technology, KIST School, Korea University of Science and Technology, Seoul 02792, Korea.

**Keywords:** MeCP2, Alzheimer's disease, cognitive dysfunctions, striatum, epigenetic regulation

## Abstract

**Rationale:** Cerebral Methyl-CpG binding Protein 2 (MeCP2) is involved in several psychiatric disorders that are concomitant with cognitive dysfunction. However, the regulatory function of striatal MeCP2 and its association with Alzheimer's disease (AD) has been largely neglected due to the absence of amyloid plaque accumulation in the striatal region until the later stages of AD progression. Considerable evidence indicates that neuropsychiatric symptoms related to cognitive decline are involved with striatal dysfunction. To this respect, we investigated the epigenetic function of striatal MeCP2 paralleling the pathogenesis of AD.

**Methods:** We investigated the brain from amyloid precursor protein (APP)/presenilin1 (PS1) transgenic mice and postmortem brain samples from normal subjects and AD patients. The molecular changes in the brain, particularly in the striatal regions, were analyzed with thioflavin S staining, immunohistochemistry, immunoblotting, and MeCP2 chromatin immunoprecipitation sequencing (ChIP-seq). The cognitive function of APP/PS1 mice was assessed via three behavioral tests: 3-chamber test (3CT), Y-maze test (YMT), and passive avoidance test (PA). A multi-electrode array (MEA) was performed to analyze the neuronal activity of the striatum in APP/PS1 mice.

**Results:** Striatal MeCP2 expression was increased in the younger (6 months) and older (10 months) ages of APP/PS1 mice, and the genome-wide occupancy of MeCP2 in the younger APP/PS1 showed dysregulated binding patterns in the striatum. Additionally, we confirmed that APP/PS1 mice showed behavioral deficits in multiple cognitive behaviors. Notably, defective cognitive phenotypes and abnormal neuronal activity in old APP/PS1 mice were rescued through the knock-down of striatal MeCP2.

**Conclusion:** We found that the MeCP2-mediated dysregulation of the epigenome in the striatum is linked to the defects in cognitive behavior and neuronal activity in the AD animal model, and that this alteration is initiated even in the very early stages of AD pathogenesis. Together, our data indicates that MeCP2 may be a potential target for the diagnosis and treatment of AD at asymptomatic and symptomatic stages.

## Introduction

Alzheimer's disease (AD) is a neurodegenerative disease which is the most common form of dementia. AD affects about 10% of people of ages 65 and older, and the number of patients is expected to grow consistently by 2050 1. However, there are few effective treatments to date that can cure or stop the progression of AD. Among the various behavioral and neuropsychiatric symptoms of AD, cognitive decline is the predominant symptom that is accompanied by pathological hallmarks such as the accumulation of extracellular amyloid β (Aβ) plaques and the intracellular neurofibrillary tangles in the brain 2.

Meanwhile, cognitive decline also manifests in other various neuropsychiatric diseases 3, 4, and the striatum has been shown to regulate this phenomena by affecting both psychiatric symptoms and cognitive decline simultaneously 5, 6. The striatum has been established as a region critical for motor and reward systems, receiving inputs from multiple reward associated neuronal circuitries and conjugates goal-driven behaviors with rewards. Additionally, the striatum has been also shown to participate in other multiple cognitive functions that can affect hippocampal memory, including action planning, decision-making, motivation, social interactions, and reward perception 7-10. In the context of AD, neuroanatomical studies of AD patients have confirmed alterations in caudate nucleus volume, a region part of the human striatum, compared to normal individuals 11, 12, which indicates that there may be an correlation between AD progression and striatal regions. Despite this link, the biological function of the striatum in AD has not yet been studied as Aβ plaque accumulation cannot be found in this region until the late stages of AD 13-16. Therefore, we hypothesized that striatum dysfunction may affect cognitive decline in AD.

Methyl-CpG Binding Protein 2 (MeCP2) is an epigenetic regulator that modulates transcription by binding to various regions of methylated loci of overall genomic DNA 17. Numerous studies have shown that the alterations in DNA methylation is critical to the aging brain and in the brain of AD patients 18-20. MeCP2 was known to play a critical role in the nervous system such as controlling the expression of BDNF 21-23 and manipulating the excitatory and inhibitory synapse 24-26. In the striatum, MeCP2 regulates dopamine content, and controls the number of interneurons and its dendritic arborization 27. Furthermore, increasing MeCP2 in the striatum causes the alteration of BDNF and results in the failure of drug intake control 28. Recent studies have suggested that MeCP2 regulates the risk genes of AD such as *MEF2C* (Myocyte Enhancer Factor 2C) 29 and* ADAM10* 30. In a mouse tauopathy model with human tau overexpression, MeCP2 was identified as a candidate protein that may regulate tau pathology in the hippocampus 31, and MeCP2 protein level was found to be increased in the cortical neurons 32. Furthermore, MeCP2 triplicate mice caused increased tau phosphorylation in the cortex and hippocampus, and resulted in excitotoxicity in the same regions 33. However, despite several reports dealing with the association between MeCP2 and AD, the exact epigenetic mechanism of MeCP2 affecting AD pathology and their interplays within the striatal region need to be further elucidated.

In this study, we analyzed the functional role of striatal MeCP2 affecting cognitive behaviors in an AD mouse model. As the striatum is one of the brain regions responsible for memory impairment, we focused on the striatum to elucidate the cause of deficits in cognitive phenotypes in the AD mice model. Consequently, we explored the molecular and functional changes of MeCP2 in the striatum along with the progression of AD-like cognitive phenotypes to propose striatal MeCP2 as a potential target for AD treatment.

## Results

### Expression of striatal MeCP2 is increased in the early (6-month-old) and late (10-month-old) stages of APP/PS1 mice

**Figure 1:**
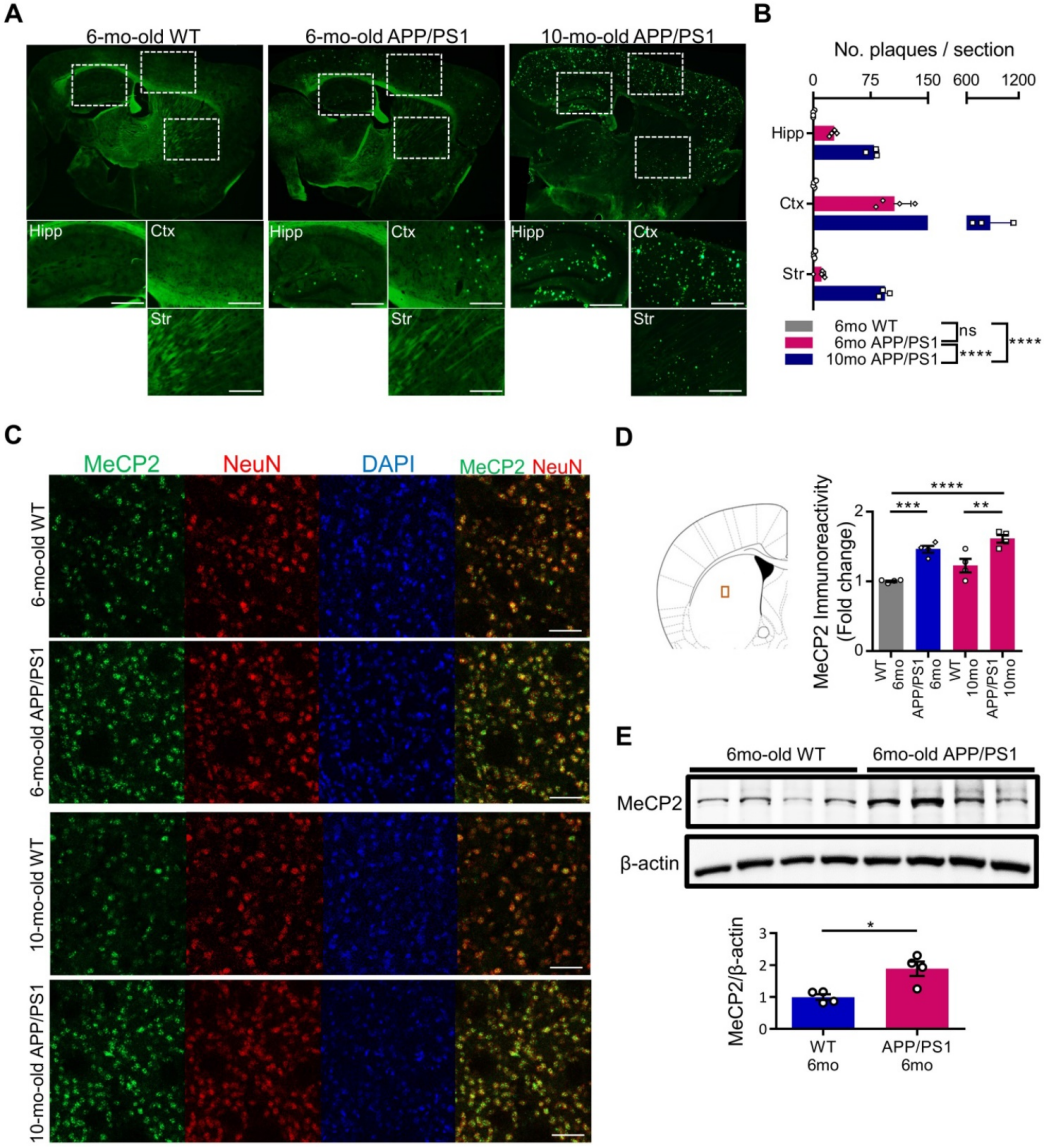
** Striatal MeCP2 expression was increased in 6-month-old and 10-month-old APP/PS1 mice. (A)** Representative images of thioflavin S staining on sagittal sections from 6-mo WT and 6,10-mo APP/PS1 mice. Distinctive bright green locales on the sections of APP/PS1 reveal Aβ plaques. Hipp: hippocampus, Ctx: cerebral cortex, Str: striatum, Scale bars: 300 µm. **(B)** 10-mo APP/PS1 showed a significant difference in the number of plaques when compared with other groups (mean ± SEM, 6-mo WT n = 4; 6-mo APP/PS1 n = 4; 10-mo APP/PS1 n = 3; ****P < 0.0001; Two-way ANOVA with Tukey post-hoc test) **(C)** Representative IHC images of MeCP2 co-stained with neuronal marker NeuN in the striatal region of 6,10-mo WT and 6,10-mo APP/PS1 showed increased levels of MeCP2 expression in the APP/PS1 mice. Scale bars: 50 µm.** (D)** Signal intensity of MeCP2 immunoreactivity was quantified in 6,10-mo WT and 6,10-mo APP/PS1 mice by neuronal marker NeuN and is displayed as fold change in MeCP2 immunoreactivity (mean ± SEM, 6-mo WT n = 4; 6-mo APP/PS1 n = 4; 10-mo WT n = 4; 10-mo APP/PS1 n = 4; **P < 0.01, ***P < 0.001, ****P < 0.0001; One-way ANOVA with Tukey post-hoc test). The microscopic area is marked by a red box to indicate the striatum. **(E)** Western blot analysis of MeCP2 protein compared to β-actin levels in the striatum of 6-mo WT and APP/PS1 mice (mean ± SEM, WT 6-mo n = 4; APP/PS1 6-mo n = 4; Student's *t*-test, *P < 0.05).

MeCP2 is a regulator of gene expression that plays an important role in the epigenetic regulation of the central nervous system. Striatal MeCP2, localized within the nucleus of the dorsal striatum region, has been involved in the regulation of neuronal activity of the striatum that is associated with reward and drug addiction-related behaviors 28. In order to identify the molecular mechanism underlying the cognitive deficits of APP/PS1 mice, we performed thioflavin S staining to confirm the presence of Aβ plaques in 6-month-old and 10-month-old APP/PS1 mice. In the 6-month-old mice, occasional Aβ plaques were observed in the hippocampus and the cortical regions of the APP/PS1 mice, while wildtype mice (WT) did not show positive signals in either of these regions. However, in 10-month-old APP/PS1 mice, significantly increased number of plaques were detected in multiple regions throughout the brain (Figure 1A-B). To explore the early molecular changes in the brain, next we examined MeCP2 expression in the striatum of the 6-month and 10-month-old APP/PS1 mice. Interestingly, we found that the neuronal MeCP2 levels within the striatal regions were significantly increased in the MeCP2 immunofluorescence detection and the western blot analysis (Figure 1C-E). Other regions including the hippocampus, habenula, and thalamus showed little difference in MeCP2 levels between the WT and APP/PS1 groups (Figure S1A-D). Despite the rare presence of amyloid plaques in the striatal region, the increase of striatal MeCP2 levels in 6-month-old APP/PS1 mice suggest that there may be noteworthy changes in epigenetic regulation by MeCP2 occurring throughout the pathogenesis of AD and the manifestation of cognitive dysfunctions in the APP/PS1 mice.

### Genome-wide occupancy of MeCP2 in early stage of APP/PS1 showed dysregulated binding patterns

**Figure 2:**
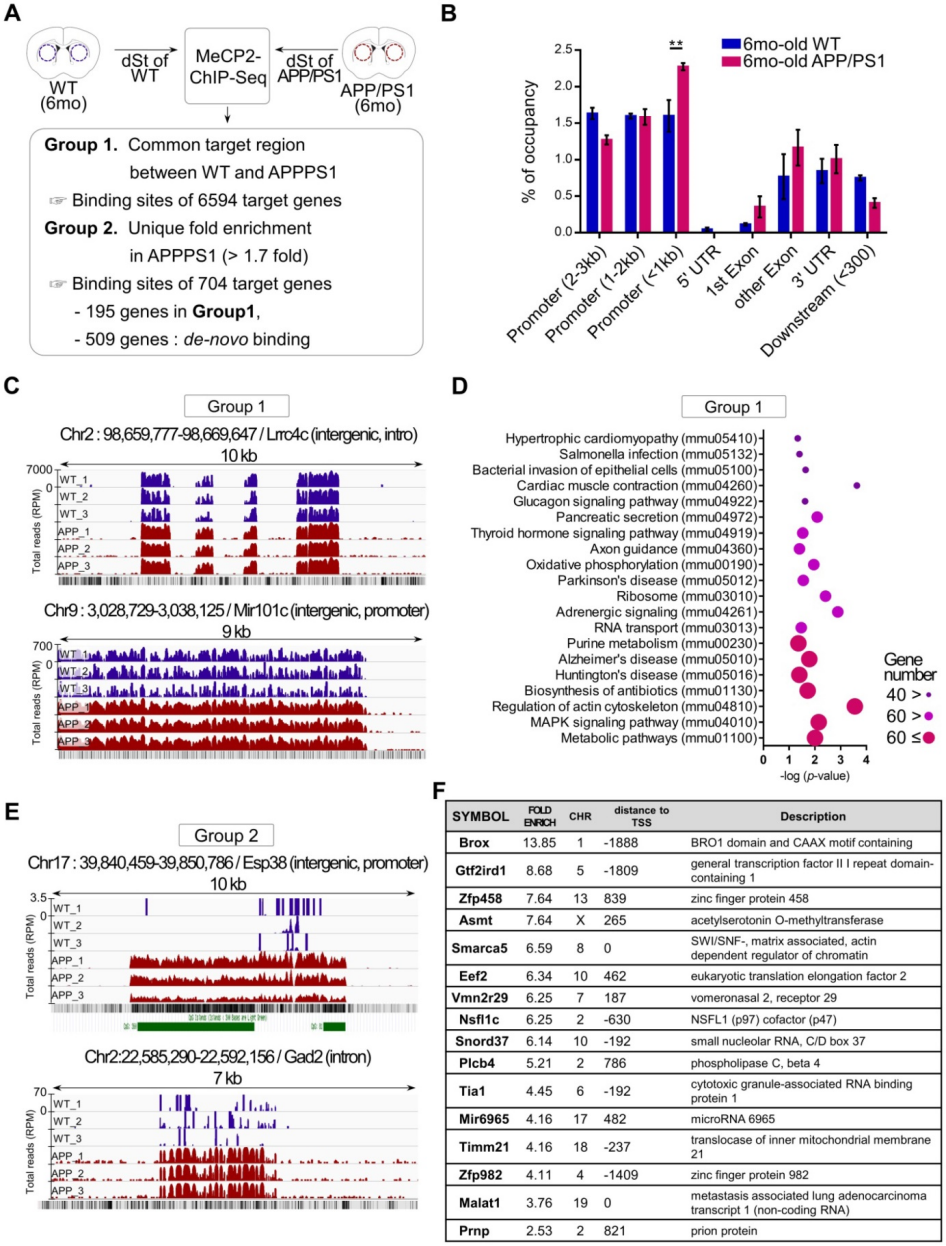
** MeCP2-ChIP-seq analysis reveals altered binding patterns of MeCP2 upon several target genes involved in the pathway of AD. (A)** Flowchart showing the schematics of MeCP2-ChIP-seq analysis and the selection criteria for group1 and group2 that resulted from ChiP-seq analysis. **(B)** Bar graphs show the coverage of bound regions of MeCP2 within WT control and APP/PS1 mice. Overall trends showed an increased binding pattern of MeCP2 in APP/PS1 mice, and the proximal promoter region (< 1kb) showed increased MeCP2 occupancy in the APP/PS1 (mean ± SEM, WT n = 3; APP/PS1 n = 3; ** *P* < 0.01). **(C)** Representative images of IGV comparison analysis from group1. WT (dark blue) peaks and APP/PS1 (red) peaks each represent an accumulation of read counts of the ChIPed-DNA from the selected region. **(D)** KEGG pathway analysis of group1 showed that the MeCP2 occupancy on the genome was involved in several biological pathways including the major neurodegenerative pathways (AD, PD, HD) and cytoskeleton regulation. **(E)** Representative images of IGV comparison analysis of group2. WT (dark blue) peaks and APP/PS1 (red) peaks each represent an accumulation of read counts of the ChIPed-DNA from the selected region. Green boxes indicate CpG island regions detected in the UCSC genome browser (RPM: reads per million). **(F)** Table of the MeCP2 major binding sites to the target genes from group2, which shows the top 16 genes in descending order of fold-enrichment along with additional information (chromosome number, distance to transcription start site, brief description of the target gene).

To better understand the functional changes driven by the increased expression of striatal MeCP2 in APP/PS1 mice, alterations in the genome-wide binding patterns of striatal MeCP2 were analyzed through MeCP2-chromatin immunoprecipitation sequencing (MeCP2-ChIP-Seq) followed by bioinformatics analysis of the MeCP2-bound genomic loci. MeCP2-ChIP-Seq analysis was performed via massive parallel 75-base pair sequencing of the ChIPed DNA samples from the striatum, and unique binding regions of MeCP2 based on data obtained from the WT and APP/PS1 groups through MAnorm comparison analysis was identified (Figure 2A). Distribution of MeCP2 genomic occupancy in the vicinity of coding sequences of genes showed increased binding ratio in the striatum of APP/PS1 mice, and the proximal promoter region (< 1kb) showed significantly increased occupancy (Figure 2B and Figure S2B). We defined the selection criteria for MeCP2 target regions and characterized two groups resulting from the initial analysis: group1 was defined as the common target regions between the WT and APP/PS1 groups, and group2 was defined as AD-specific target regions selected on the basis of > 1.7-fold enrichment specific to the APP/PS1 group compared to the WT group. Group2 target regions with increased binding levels in the APP/PS1 group were composed of the 195 genes from group1 and *de novo* target regions of 509 genes discovered from the read sequences were obtained from the APP/PS1 group. Representative images from group1 and group2 displayed through the Integrative Genomics Viewer (IGV) analysis showed different binding patterns of MeCP2 within the APP/PS1 group. Increased binding levels were found in group1, and *de novo* binding was found in the APP/PS1 mice of group2 (Figure 2C, E and Figure S2C). KEGG (Kyoto Encyclopedia of Genes and Genomes) pathway analysis of the common targets from group1 revealed that several important pathways of AD were occupied and regulated by MeCP2 throughout the genome (Figure 2D). The IGV images from group2 showed that the read counts of the ChIP-DNA from the selected regions were significantly altered in the APP/PS1 mice, while the WT mice did not show as many occupancy patterns on the GC-rich regions or expected CpG islands (Figure 2E). Furthermore, a list of selected target regions from group2 included genes implicated in neuronal functions (Figure 2F). 704 genes within group2 occupied various KEGG pathways and gene ontology (GO) terms that were associated with crucial neuronal functions (Figure S2A, D). In addition, we confirmed the binding of the target regions through ChIP-qPCR (Figure S2E). The changes in the expression of several MeCP2 target regions were confirmed through qPCR analysis using the striatal RNA samples (Figure S2F-G). We reverified these genes by searching for previously established studies that investigated these genes in relation to AD pathology and neuronal function in the brain (Figure S2H). These results indicate that MeCP2 may regulate pathways implicated in AD pathogenesis, and the dysregulated *de-novo* patterns of striatal MeCP2 genomic occupancy during the early ages of APP/PS1 mice may contribute to various molecular changes in the early AD phenotypes.

### APP/PS1 mice shows behavioral deficits in multiple cognitive behaviors

**Figure 3:**
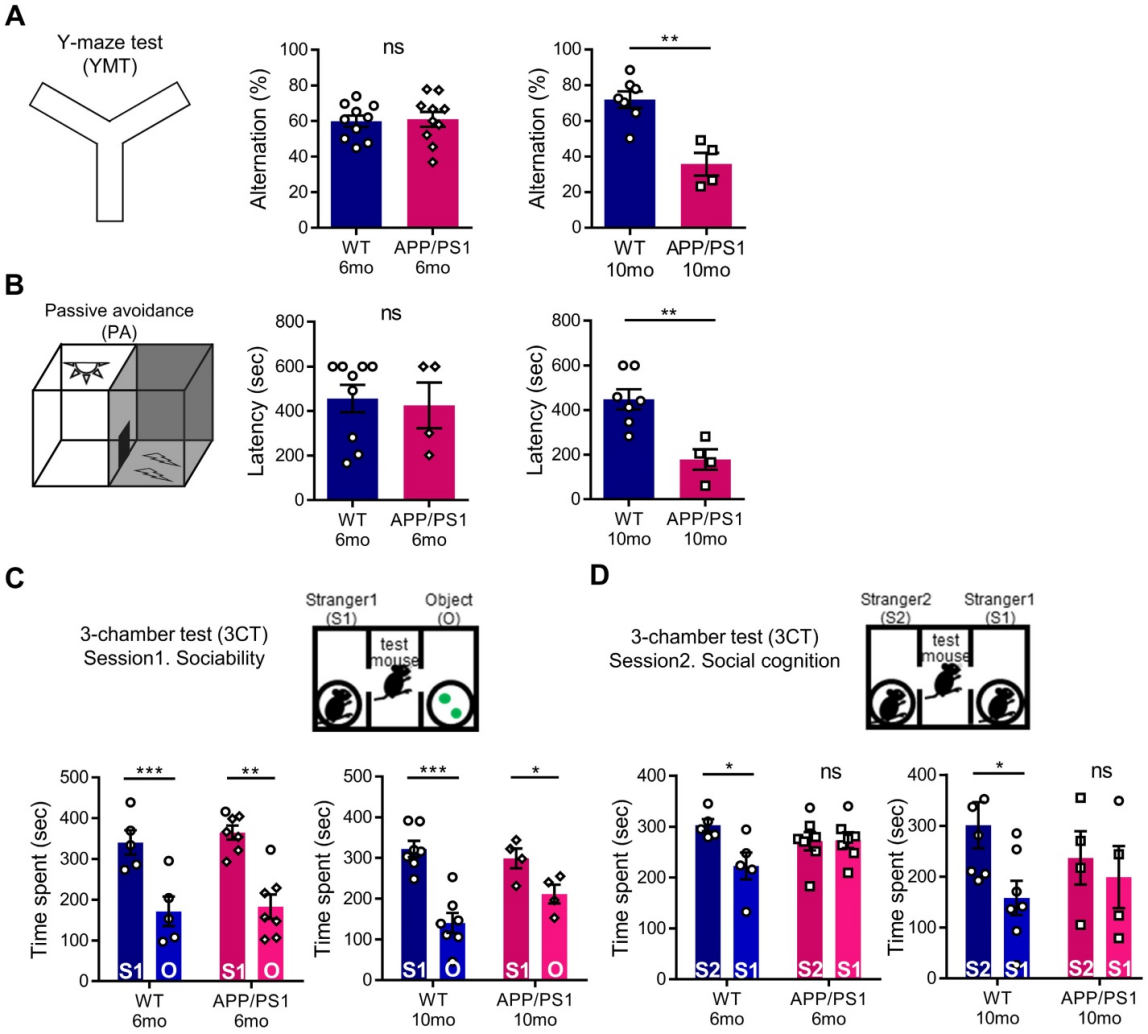
** APP/PS1 mouse showed behavioral deficits in multiple cognition tests. (A)** Memory function of WT and APP/PS1. Alternations in YMT arm exploration was indicated by percentage alternation (mean ± SEM, 6-mo WT n = 10; 6-mo APP/PS1 n = 10; 10-mo WT n = 7; 10-mo APP/PS1 n = 4; Student's *t*-test, **P < 0.01). **(B)** In the PA test, time elapsed to escape the dark chamber is shown as latency in seconds (6-mo WT n = 9; 6-mo APP/PS1 n = 4; 10-mo WT n = 7; 10-mo APP/PS1 n = 4; Student's *t*-test, **P < 0.01). **(C)** Sociability is shown in the first session of 3CT test of the APP/PS1 and WT groups (mean ± SEM, 6-mo WT n = 5; 6-mo APP/PS1 n = 7; 10-mo WT n = 7; 10-mo APP/PS1 n = 4; Student's *t*-test, *P < 0.05, **P < 0.01, ***P < 0.001).** (D)** Social cognition is shown in the second session of the 3CT of APP/PS1 and WT mice (mean ± SEM, 6-mo WT n = 5; 6-mo APP/PS1 n = 7; 10-mo WT n = 7; 10-mo APP/PS1 n = 4; Student's *t*-test, *P < 0.05).

To evaluate abnormal behavioral changes preceding the general cognitive dysfunction of APP/PS1 mice, we conducted a behavior assessment battery on 6-month-old and 10-month-old mice. As expected, in APP/PS1 mice of 6 months, which show initial amyloid deposition with preserved memory function 34, the amyloid load shown in Figure 1 was not yet associated with hippocampal memory function deficits. In the YMT and PA tests, similar to previous studies, 6-month-old APP/PS1 mice showed a preservation of learning and memory, whereas the 10-month-old mice manifested cognitive defects (Figure 3A-B) 35, 36. To further analyze the changes in the cognitive phenotype, we performed the 3CT to determine alterations in social memory by measuring for sociability and social cognition in the APP/PS1 and WT mice. Both the WT and APP/PS1 groups did not show a preference for a specific testing chamber during the habituation stage. In the sociability session, both WT and APP/PS1 mice showed similar levels of social activity and spent significantly more time in the social chamber (S1) compared to the object chamber (O), indicating intact sociability in APP/PS1 mice (Figure 3C). Interestingly, in the subsequent social cognition session, APP/PS1 mice did not prefer either chamber, while the WT mice were able to discriminate between the familiar (S1) and the novel mouse (S2) chambers (Figure 3D). The APP/PS1 mice displayed significantly diminished social memory function compared with the WT group and these social memory alterations manifested similarly in both 6-month and 10-month-old APP/PS1 mice (Figure 3D). Similar levels of total entry frequency between the two groups in the sociability and social cognition sessions indicated that there was no change in their exploratory behavior during the testing sessions. Taken together, these results indicate that general cognitive deficits manifest in the late stage of the APP/PS1 mice, and that social memory impairment is also observed prior to the appearance of general cognitive dysfunction in the early stages of the APP/PS1 mice.

### Defective cognitive behaviors and abnormal neuronal activity of late-stage APP/PS1 were rescued through the knock-down of striatal MeCP2

**Figure 4:**
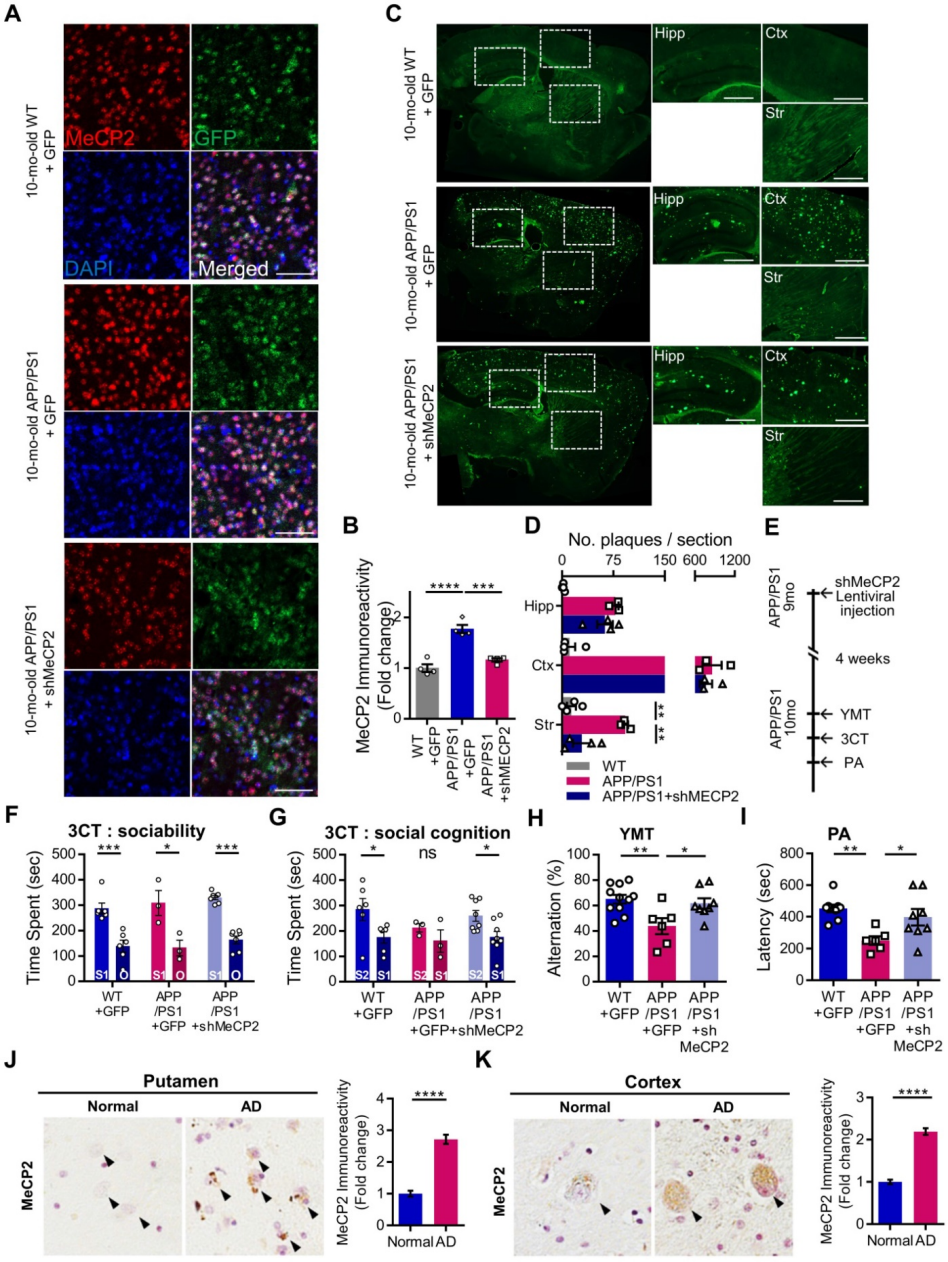
** 10-month-old APP/PS1 with impaired memory also manifested defects in social cognition and knock-down of striatal MeCP2 rescued both cognitive deficits. (A)** Representative IHC staining images of MeCP2, GFP, and DAPI from the striatal region of 10-mo WT with GFP virus injection (WT+GFP) and 10-mo APP/PS1 with GFP virus injection (APP/PS1+GFP) compared to APP/PS1 with shMeCP2 virus injection (APP/PS1+shMeCP2). Scale bars: 50 µm **(B)** Signal intensity of MeCP2 immunoreactivity was quantified in WT+GFP and APP/PS1+GFP compared to APP/PS1+shMeCP2 as fold change in MeCP2 immunoreactivity (mean ± SEM, n = 4 each; ***P < 0.001, ****P < 0.001; One-way ANOVA with Tukey post-hoc test). **(C)** Representative thioflavin S staining images of sagittal sections. Distinctive bright green locales on sections from APP/PS1 mice reveal Aβ plaques. Hipp: hippocampus, Ctx: cerebral cortex, Str: striatum, Scale bars: 300 µm **(D)** Number of plaques in each region (mean ± SEM, WT+GFP n = 4; APP/PS1+GFP n = 4; APP/PS1+shMeCP2 n = 4, **P < 0.01). **(E)** Experimental schedule of behavioral tests after stereotaxic injection of shMeCP2 virus. Tests were conducted 4 weeks after the surgery. **(F)** Time spent in chambers shown as a measure of sociability in the first session of the 3CT of WT+GFP, APP/PS1+GFP and APP/PS1+shMeCP2 groups. **(G)** Time spent in chambers as a measure of novelty-seeking in the second session of 3CT of WT+GFP, APP/PS1+GFP and APP/PS1+shMeCP2 groups (for (F) and (G), mean ± SEM, WT+GFP n = 6; APP/PS1+GFP n = 3; APP/PS1+shMeCP2 n = 8; Student's *t*-test, *P < 0.05, **P < 0.01, ***P < 0.001). **(H)** Memory function of WT+GFP, APP/PS1+GFP, and APP/PS1+shMeCP2 groups. Alternations in YMT arm exploration is indicated by percentage (mean ± SEM, WT+GFP n = 11; APP/PS1+GFP n = 6; APP/PS1+shMeCP2 n = 8; *P < 0.05, **P < 0.01). **(I)** In the PA test, time elapsed to escape the dark chamber is shown as latency in seconds (WT+GFP n = 9; APP/PS1+GFP n = 7; APP/PS1+shMeCP2 n = 8; *P < 0.05, **P < 0.01). **(J)** The immunoreactivity of MeCP2 in the putamen of AD postmortem brain compared to normal subject (Normal). Arrowheads (black) indicate MeCP2-positive neurons. Densitometry analysis showing the fold change of MeCP2 expression in the putamen of AD postmortem brains compared to brains from normal subjects (brown MeCP2-positive cells with purple hematoxylin counter staining, number of subjects (normal/AD) per group: N = 3, cell counts: n = 30 for each group (10 cells/1 subject); Student's *t*-test, ****P < 0.0001). **(K)** The immunoreactivity of MeCP2 in the temporal cortex of AD postmortem brains compared to brains from normal subjects (Normal). Arrowheads (black) indicate MeCP2-positive neurons. Densitometry analysis showing the fold change of MeCP2 expression in the temporal cortex of AD postmortem brains compared to brains from normal subjects. MeCP2 immunoreactivity was elevated within the nucleus and, partially, in the cytosolic compartment of neurons (brown MeCP2-positive cells with purple hematoxylin counter staining, number of subjects (normal/AD) per group: N = 3, cell counts: n = 30 for each group (10 cells/1 subject); Student's *t*-test, ****P < 0.0001).

**Figure 5:**
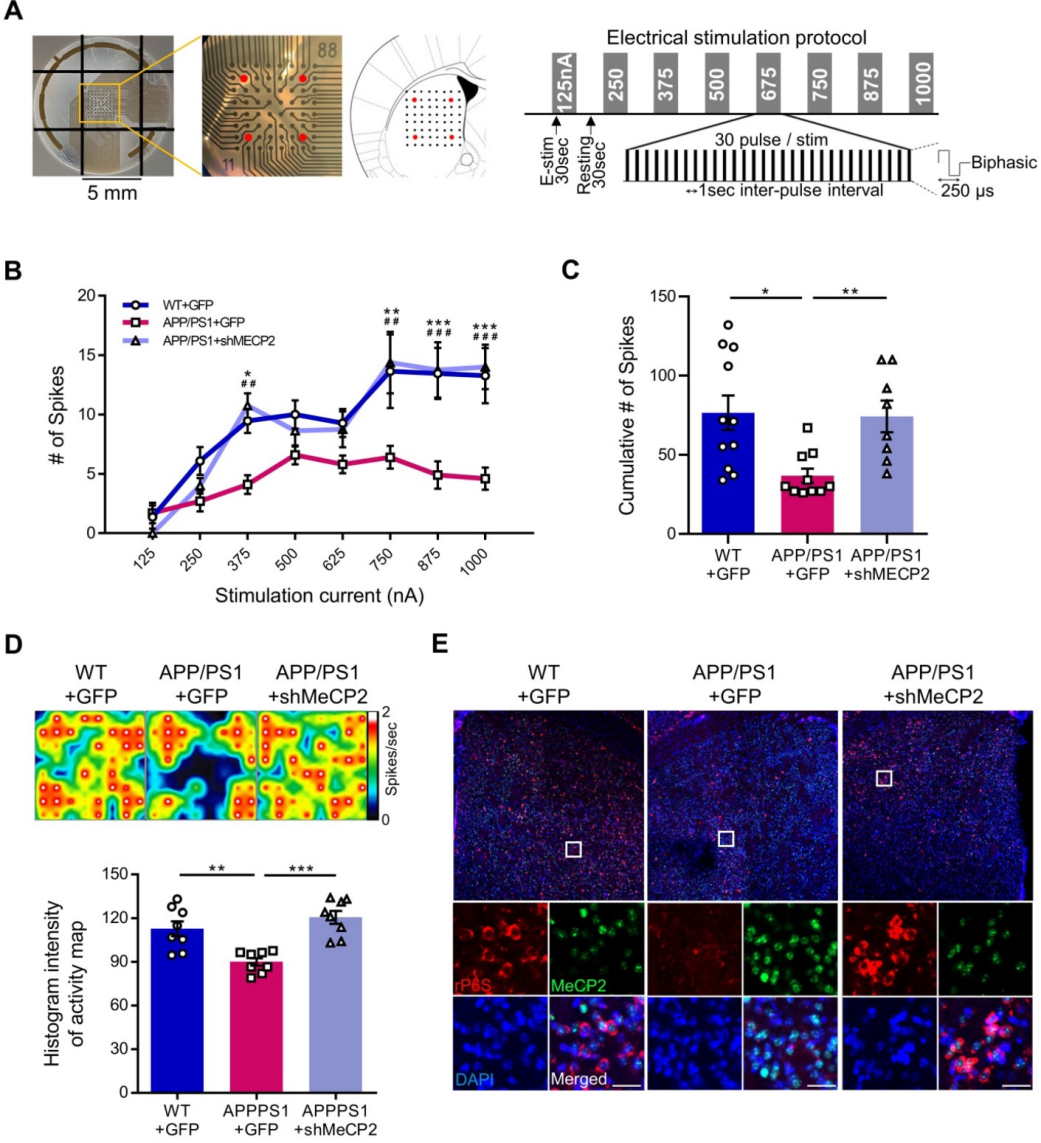
** Knock-down of MeCP2 expression in the striatum rescued the neuronal activity of late-stage APP/PS1 mouse (10-month-old). (A)** Diagram showing 4 stimulation positions (red dots) of the total 64 probes in each well and protocol summary of MEA experiment showing the stepwise increasing stimulation procedures **(B)** Line plot showing the number of stimulated firings of the striatal neurons after each stimulation current. High-current (750, 875, 1000 nA) stimulations showed significantly reduced firing in the APP/PS1+GFP group and rescued neuronal activity pattern in the knockdown group (mean ± SEM, n = 11, 10, 8 cells from 2 mice, respectively, *P < 0.05; **,##*P* < 0.01; ***,###*P* < 0.001; Asterisk indicates statistical significance between WT+GFP and APP/PS1+GFP; sharp indicates statistical significance between APP/PS1+GFP and APP/PS1+shMeCP2). **(C)** Bar graphs showing the cumulative number of firings from all stimulation currents (mean ± SEM, n = 11, 10, 8 cells from 2 mice, respectively, *P < 0.05, **P < 0.01). **(D)** Representative heatmap image and quantification (Histogram densitometry) bar graph of neuronal activity on the MEA probes. APP/PS1+MeCP2 knockdown group showed rescued patterns (n = 8, respectively, **P < 0.01, ***P < 0.001).** (E)** MeCP2 IHC staining (green) with rP6S (red) neuronal activity marker staining in the striatal region of WT, APP/PS1 and APP/PS1 with MeCP2 knockdown groups. Scale bars: 30 µm.

We used 10-month-old APP/PS1 mouse to confirm the effects of striatal MeCP2 increase via virus-mediated knock-down experiments. To elucidate the consequences of genetic manipulation leading to the increase in striatal MeCP2 levels, we injected lentiviral short hairpin RNA of MeCP2 (shMeCP2) to knockdown the expression of MeCP2 in the striatal regions of APP/PS1 mice. Increased level of MeCP2 signal was also detected in the striatal region of APP/PS1+GFP group and MeCP2 expression downregulation was observed in the APP/PS1+shMeCP2 group compared to the WT group (Figure 4A-B). Coinciding with the downregulation of MeCP2, the number of Aβ plaques shown by thioflavin S staining decreased significantly in the striatum of the knockdown group compared to the APP/PS1+GFP group (Figure 4C-D). Interestingly, in the 3CT, YMT, and PA tests performed 4 weeks after the injection (Figure 4E), previously observed cognitive dysfunctions were simultaneously rescued by the knock-down of MeCP2 expression (Figure 4F-I). In the 3CT sociability session, shMeCP2-injected APP/PS1 mice showed a normal pattern of exploring (Figure 4F). Notably, the shMeCP2 injection rescued social memory deficits to the level of their WT littermates (Figure 4G). Additionally, the cognitive deficits in the YMT and the PA test were also rescued by MeCP2 knockdown (Figure 4H-I). Furthermore, when compared to the WT group, the APP/PS1 and APP/PS1 with shMeCP2 virus injection groups did not show significant changes in locomotion and motor learning in the 3CT and rotarod tests (RTT), indicating that neuropathology did not sufficiently progress (Figure S3A-D). In addition, we tested an amyloid-positive acute AD mouse model which elicited AD-like cognitive symptoms following the intracerebroventricular stereotaxic injection of Aβ_1-42_ oligomer solution (ICVA) 37. After ICVA, the ICVA model showed memory deficits in the PA test (Figure S3E-G). Immunostaining results also showed increased MeCP2 signals in the striatal region of the ICVA mice (Figure S3H). 3CT behavioral phenotypes of social cognition were rescued in the ICVA shMeCP2 knock-down mice (Figure S3I-L). This suggests that increased MeCP2 levels in the striatum of the ICVA mouse model contributes to deficits in multiple cognitive phenotypes and that these defective behaviors can be rescued by the knock-down of striatal MeCP2 expression. Additional evidence from AD patients showed that there is an aberrant MeCP2 expression pattern in the putamen beyond the initial stages of AD, in which a significant increase in MeCP2 level was found in the postmortem brains of AD patients compared to healthy individuals. To examine the localization of MeCP2 and its immunoreactivity in normal individuals and AD patients, we performed immunohistochemistry and verified MeCP2 immunoreactivity in the putamen and the cortex of postmortem brains. MeCP2 immunoreactivity was elevated within the nucleus and partially in the cytosolic compartment of neurons of AD patients when compared to normal individuals. Densitometry analysis showed significantly increased MeCP2 levels both in the putamen and temporal cortex of AD postmortem brains (Figure 4J-K). This data coincided with the western blot analysis, indicating increased MeCP2 levels in the postmortem forebrain regions of AD patients (Figure S3M and Table S1). Furthermore, cortical regions of the late-stage APP/PS1 mice also displayed increased levels of MeCP2 (data not shown). To test the neuronal activity of the striatum, brain slices of the striatum regions were analyzed on the MEA with 64 electrodes in each well (Figure 5A). Knock-down of MeCP2 expression in the striatum rescued the altered neuronal activity in the late stages of symptomatic 10-month-old APP/PS1 mice. The number of stimulated firings of striatal neurons following stimulation currents with stepwise increments showed a pattern of neuronal activity that was significantly rescued in the high-current stimulated knock-down group (Figure 5B-C). Representative heat-map images of neuronal activity from the MEA probes showed rescued firing patterns in the APP/PS1+MeCP2 knock-down group (Figure 5D). MeCP2 co-stained with Ribosomal Protein S6 (rP6S), a marker for neuronal activity, also indicated significantly rescued neuronal activity in the knock-down group (Figure 5E). Altogether, these findings provide evidence that increased MeCP2 levels in the early stages of AD may be maintained within the brain until the later stages of the AD, and that several defective phenotypes in the amyloid-positive symptomatic mouse model may be rescued through the knock-down of striatal MeCP2.

## Discussion

In the present study, we observed the association of striatal MeCP2 with the manifestation of multiple cognitive phenotypes including general hippocampus-dependent memory impairment and social memory dysfunction in the early and late stages of AD. Striatal activity is particularly critical for controlling cognitive dysfunctions in various brain disorders including AD. Therefore, we hypothesized that striatal activity, when controlled by striatal MeCP2, may be key in discovering potential therapeutic targets which have been altered at the beginning of amyloid load accumulation. We have demonstrated that the 6-month old APP/PS1 mice show Aβ plaques while in absence of hippocampal memory abnormalities, as established in previous studies 34, 38, 39. However, we have observed alterations in social memory in amyloid-positive APP/PS1 mice. These deficits were consistently observed in the symptomatic stages of older APP/PS1 mice at 10 months of age and in the ICVA mice model, which exhibits hippocampal memory decline as shown in Figure 3 and Figure S3. Additionally, we confirmed social memory impairments in the early amyloid-positive stages which lasted into the later ages of the APP/PS1 mice. Furthermore, we found significantly increased MeCP2 levels in the striatum of APP/PS1 mice and ICVA mice. Consequently, alterations in MeCP2 expression may be a major cause of cognitive decline. Meanwhile, we observed that viral shRNA-mediated knock-down of striatal MeCP2 rescued several cognition deficits. To identify the alterations in genome-wide binding patterns of the striatal MeCP2 in APP/PS1 mice, we performed MeCP2-ChIP-seq. Intriguingly, MeCP2-ChIP-seq analysis revealed severely dysregulated MeCP2 binding patterns, and bioinformatics analysis highlighted abnormal patterns that were observed in several genes that make up the KEGG pathways of AD.

Owing to the absence of plaque accumulation in the striatum until the late stages of AD, little is known about the regulation of striatal activity and the striatal MeCP2 function in AD pathogenesis. Interestingly, in clinical studies involving AD patients, the association between the change in caudate putamen volume and AD was reported 11, 12, and studies have reported that the change in the striatal function in AD animal models and patients may induce anxiety and depressive-like symptoms 40, 41. In line with these reports, we confirmed that cognitive dysfunctions, a major behavioral symptom of AD, can be improved by regulating striatal function through MeCP2 manipulation.

Furthermore, we also found that the late stage APP/PS1 mice and the ICVA mouse model, which exhibited hippocampal memory decline 37, showed increased expression of MeCP2 in the striatum compared to control mice. Surprisingly, we found that the downregulation of MeCP2 in the APP/PS1 and ICVA mice rescued the deficits in cognitive phenotypes. To our knowledge, this is the first study that explores striatal MeCP2 as a novel AD target throughout the stages of AD, from the amyloid-positive asymptomatic stage to the phase when multiple memory declines occur. Furthermore, we confirmed that the decrease in striatal neuronal activity in the late stages of APP/PS1 mice was restored through MeCP2 knockdown, which was consistent with the previous report describing the association between striatum function and the regulation of cognitive function 5, 6. Over the past decade, several studies investigating striatal involvement in cognitive function have emerged, detailing the hippocampal-striatal communication based on the interplay between the neocortical-basal ganglia circuit and the temporal lobe-hippocampal system in mice and humans 42-44. Although the current study directly manipulated the expression of MeCP2 and confirmed the recovery of neuronal activity specific to the striatal regions and not in the hippocampus, we found that the manipulation of striatal MeCP2 recovered cognitive deficits related to hippocampal memory in the later stages of neuropathology. Through this, we confirmed that MeCP2 knockdown was sufficient to restore cognitive function through the recovery of striatal function in the APP/PS1 mouse model, and this suggested that striatal MeCP2 may have a therapeutic effect in the pathogenesis of AD.

Another novel point of our study is that we observed altered binding patterns of striatal MeCP2 in the early stages of APP/PS1 mice. MeCP2 ChIP-seq analysis revealed severely dysregulated binding patterns of MeCP2 in APP/PS1 mice. The abnormal binding pattern of MeCP2, as a transcriptional regulator, may have played a role in affecting AD pathology. In normal physiological conditions, MeCP2 inhibits transcription by histone deacetylation 45, 46 or methylation 45-47. In addition, MeCP2 also regulates transcriptional activation by association with the transcriptional activator CREB1 at the promoter region of an activated target 23. Previous studies have demonstrated that MeCP2 mutation results in impaired MeCP2 binding to methylated DNA, leading to the development of Rett syndrome-like phenotypes in mice models 48, 49. Thus, the abnormal binding pattern of MeCP2 in APP/PS1 mice may cause impaired regulation of the genes responsible for AD pathology, thereby leading to perturbation in the expression of these genes. Interestingly, our study demonstrated that within APP/PS1 mice, altered binding patterns of MeCP2 target genes were included within the KEGG pathways or GO terms of striatal activity (Figure S2D). Among these genes, *PRNP, SMARCA5, EEF2 and PLCB4* have been suggested as having crucial roles in AD pathogenesis 50-56. Dysregulated binding of MeCP2 on these genes lead to the progression of AD pathology.

Furthermore, increased binding of MeCP2 may also result in the abnormal expression of various kinds of genes including AD risk genes. Our unpublished MeCP2 binding analysis revealed increased binding of MeCP2 to *MEF2C* and *ADAM10*. In the largest GWAS of late-onset Alzheimer disease to date, *MEF2C* and *ADAM10* were identified as risk genes for AD 57, 58. MEF2C plays a critical role in the refinement of synaptic connectivity 59, and ADAM10 functions as an α-secretase which may harbor neuroprotective function in AD 60. Interestingly, studies have revealed that MEF2C and ADAM10 are inhibited by MeCP2 23, 29, 30. We presume that the increased binding of MeCP2 in *MEF2C* and *ADAM10* may lead to hippocampal memory deficits in the APP/PS1. In addition, our MeCP2 ChIP-seq analysis also found increased binding of MeCP2 to the STriatal-Enriched Protein Tyrosine Phosphatase (*STEP*), of which its absence triggers disrupted social memory retention and social patterns 61. Thus, we suggest that increased binding of MeCP2 on the *STEP* gene may result in abrupt social memory deficits in the early ages of APP/PS1 mice. Consequently, increased MeCP2 levels may facilitate MeCP2 binding on these genes, contributing to the exacerbation of cognitive symptoms. Additionally, our study showed that the striatal MeCP2 knockdown rescue these deficits (Figure 4 and Figure 5). Based on our findings, we suggest MeCP2 as a novel therapeutic target that ameliorate social cognition deficits shown in the early stages of AD. Furthermore, by diminishing the expression of MeCP2, we may be able to hold early symptoms of AD in check and slow down the progression of AD. Studies are needed to further discover the effects of MeCP2 as an epigenetic regulator in the AD afflicted brain, non-specific to the striatal regions.

## Methods

### Animals

Male APPswe/PSEN1dE9 (line 85) transgenic mice with C57BL/6;C3H background from Jackson Laboratory (USA, stock number 004462) and the littermate wildtype (WT) mice were used. For IVCA, two-month-old C57BL/6 WT mice were used. All mice were placed in group housing on a reverse 12-hr day-night cycle with regulated temperature and humidity. All mice were supplied water and food, ad libitum. All experimental procedures including the usage and treatment of animals were approved by Animal Care and Use Committee of Korea Institute of Science and Technology (KIST).

### Human postmortem brain samples

Neuropathological processing of postmortem brain samples from normal subjects and AD patients was performed using procedures previously established by the Boston University Alzheimer's Disease Center (BUADC) 62. All brains were donated with consent of the next of kin after death. Institutional review board approval was obtained through the BUADC. The study was performed in accordance with institutional regulatory guidelines and principles of human subject protection in the Declaration of Helsinki. Detailed information of brain tissues is described in Supplementary Table 1.

### Behavior tests

#### Passive avoidance test

The PA test was used to measure aversive stimuli-driven hippocampal and amygdala dependent memory and learning. The PA apparatus (40 cm length × 20 cm width × 20 cm height) consisted of a dark chamber and a chamber brightened by a light source. Mice were initially placed in the brightened chamber and after 1 min, the guillotine door separating the brightened and black chambers was removed, and mice were allowed to freely explore both chambers. Once mice entered the black chamber, the door was closed, and a single foot shock (0.45 mV, 2 s duration) was administered. After 24 hr, mice were placed again in the brightened room for 1 min and the door was opened. In absence of electric foot shocks, the entering latency in s was recorded to analyze avoidance behavior for a maximum of 10 min. All chambers were cleaned with 70% ethanol after each session.

#### Y-maze test

YMT was used to assess short-term spatial memory. The YMT apparatus is a black maze with three arms (35 cm length × 10 cm width × 10 cm height) positioned 120° from each other. The YMT was performed by placing the mice in the center of Y‐maze and mice were allowed to freely explore the Y‐maze for 10 min. The entry sequence to each arm was recorded and analyzed to calculate alternation percentage. Every entry was counted as valid only when all four limbs were placed in the arm.

#### Three-chamber test

To assess sociability and social memory, the 3CT, utilizing a Plexiglass box with two dividers each with openings (62 cm length × 41 cm width × 21.5 cm height), was conducted. During the habituation phase, mice were allowed to explore all 3 chambers for 10 min to determine bias for a particular chamber. In the testing phase, the mouse was placed in the middle chamber, followed by two plastic objects (O) and a stranger (S1) mouse placed into vertical metal wire cages on each side of the chambers. In the first session testing for sociability, the mouse was allowed to freely explore the three chambers for a total of 10 min. In the second session testing for social cognition, S1 was moved to the chamber opposite to the previous placement and a new stranger mouse (S2) was placed in the chamber that previously held S1. In this session the mouse was tested additionally for 10 min. The time spent in each chamber and the number of entries into each chamber were recorded and analyzed using EthoVision XT 11.5.

#### Rotarod test

To assess motor learning ability, RTT was performed with the rotarod apparatus consisting of a 35 cm long rod with a diameter of 3.7 cm placed 17 cm above the base of the apparatus. Mice were placed on the rotarod and tested at 4 rpm for 30s. After accelerating the rotation from 4 to 40 rpm over 300 s, the latency to fall from the rotarod and the rpm at the time of falling were recorded and analyzed by averaging the data from the 3 trials recorded from the 3 testing days. Trials were conducted for 3 consecutive days with 1 hr inter-trial intervals.

### Thioflavin S staining

1% Thioflavin S solution was prepared with 80% ethanol dilution. PBS-washed fixed brain slices were mounted on silane-coated slide glass (Micro Slides, MUTO GLASS) and dried. Sections were serially incubated in 70% ethanol, 80% ethanol and thioflavin S solution. Stained sections were then washed with 80% and 70% ethanol. Sections were washed again by briefly dipping twice in distilled water.

### Immunohistochemistry

Mice were anesthetized with Avertin (250 mg/kg, Sigma) and perfused with fixed ice-cold 10% formalin solution (Sigma). Post-fixation with 10% formalin solution and dehydration with 30% sucrose were continued at 4 °C. Forty μm brain sections were incubated with primary antibodies (rabbit anti-MeCP2, 1:250, 07-013, Millipore; rabbit anti-Phosph-S6 ribosomal protein, 1:250, 2211S, Cell Signaling; mouse anti-NeuN, 1:250, mab377, Millipore) and secondary antibodies (Alexa Fluor 488 goat anti-rabbit IgG, 1:400, Life technologies; Alexa Fluor 488 goat anti-mouse IgG, 1:400, Life technologies). Zeiss LSM800 Confocal Microscope was used for fluorescence microscopy. To validate the efficacy of shMeCP2, GFP-positive cells were also observed with the confocal microscope. For postmortem tissues, immunohistochemical detection of MeCP2 was performed on paraffin sections of basal forebrain tissues from fixed normal and AD postmortem brains. Briefly, paraffin embedded sections were deparaffinized, rehydrated, and treated with 3% H_2_O_2_ for antigen retrieval 62. After blocking with TBS-T containing 5% fetal bovine serum for 1 hr, sections were then incubated overnight in TBS-T bovine serum and mouse anti-MeCP2 (1:50; sc-137070, Santa Cruz). Following incubation, sections were rinsed three times with TBS-T, then incubated in a goat anti-mouse peroxidase-conjugated secondary antibody containing TBS-T to detect MeCP2 for 2~3 hr. The sections were then rinsed three times in TBS-T and further incubated in TBS-T with ABC solution (Vector Kit) for 1 hr. Antibody complexes were visualized by using diaminobenzidine and analyzed by a bright field microscopy at various magnifications.

### Western blot

To visualize the expressional changes of MeCP2, blotted membranes were blocked with using TBS-T with 5% BSA solution. Membranes were incubated with primary antibodies (rabbit anti-MeCP2 polyclonal antibody, 1:750, 07-013, Millipore; mouse anti-β actin, 1:1000, sc-47778, Santa Cruz) overnight at 4 °C. Then membranes were treated with secondary antibodies (donkey anti-rabbit IgG-HRP, 1:2000, sc-2317, Santa Cruz; donkey anti-mouse IgG-HRP, 1:2000, sc-2318, Santa Cruz) in room temperature for 2 hr, followed by visualization with a chemiluminescent substrate (SuperSignal West Pico, Thermo Scientific, MA, USA). Signals were visualized using the ImageQuant LAS4000 (GE Healthcare Life Sciences, BUX, UK) and ImageJ software was used to measure the densitometry of the bands. For the postmortem tissues, lysates were obtained from frozen normal and AD postmortem brain tissues by using cold PBS with added 100 mM Tris (pH 7.4) buffer containing 1% Triton-X 100, 150 mM NaCl, 1 mM sodium orthovanadate, 5 mM sodium fluoride, 3 mM PMSF, 3 mM DTT, 0.5 μg/mL leupeptin, and 10 μg/mL aprotinin. Twenty μg of protein was subjected to SDS-PAGE (10%) and transferred to nitrocellulose membrane (Bio-Rad). Mouse anti-MeCP2 (sc-137070, Santa Cruz) was diluted at 1:500 in TBS-T containing 1% milk and exposed to membranes overnight at 4 °C. Protein loading was controlled by probing for β-Actin (a1978, Sigma) on same membrane. Immunoreactive proteins were detected according to the enhanced chemiluminescent protocol (34096, ThermoFisher).

### ICVA model and amyloid beta silver staining

For ICVA injection, Aβ monomers were made with 1 mM Aβ dimethylsulfoxide (DMSO) stock and diluted to 10-fold in phosphate buffered saline (PBS) (100 µM Aβ, 10% DMSO, 90% PBS). After dilution, tubes were incubated at 37 °C for 3 hr. PBS solution with 10% DMSO was used as a control and vehicle for Aβ intracerebroventricular (ICV) injections. Five μL of aggregated Aβ_1-42_ was injected into the ICV region of mouse brain. Behavioral tests were performed 3 days post-injection. Aβ solutions were loaded on the 10% polyacrylamide gel to verify oligomerization of Aβ monomers. Gels were then fixed with 40% ethanol, 10% acetic acid solution and sensitized with 0.02% sodium thiosulfate. 0.1% silver nitrate solution was used for the staining.

### Lentivirus packaging and stereotaxic surgery

For the lentiviral mediated delivery of knock-down construct of the MeCP2 into the dorsal striatum, mice were anesthetized with a cocktail of ketamine (120 mg/kg) and xylazine (6 mg/kg). Mice then underwent stereotaxic surgery (Kopf, CA, USA). ShRNA against MeCP2 (NM_001081979.1, MSH026376-HIVmU6), shRNA scrambled control was packaged into pseudo-lentivirus particles following the manufacturer instructions (EndoFectin, EFL-1001-01, Genecopoeia). For each injection site, 0.5 μL of either lentiviral MeCP2 or control vector was injected bilaterally using the Hamilton syringe with a 33- gauge needle (WPI) at a rate of 0.05 μL/min into the striatum [AP + 0.98, ML ± 1.50, and DV -3.25, -3.5, -3.75 (from dura)] as according to *The Mouse Brain in Stereotaxic Coordinates* 63. After 3 weeks post-microinjection of lentiviral shMECP2, ICVA surgery was performed, and behavioral tests were conducted 3 days after the injection.

### MeCP2 ChIP-sequencing analysis

#### Chromatin Immunoprecipitation

ChIP was performed on frozen microdissected striatum from WT and APP/PS1 mice. Chromatin was crosslinked and sheared using a bioruptor at high sonication intensity for 30 m (30 s on/30 s off). Proper fragment size was verified with an Agilent 2100 bioanalyzer. Sheared chromatin was incubated overnight with the MeCP2 antibody previously bound to pre-cleared protein A/G agarose (Sigma-Aldrich).

#### Library preparation and sequencing

Methylated DNA Enrichment kit (New England Biolabs, UK) was performed using 2 μg of the sonicated DNA. The construction of the library was performed using the NEBNext® Ultra^TM^ DNA Library Prep Kit for Illumina (New England Biolabs, UK) according to manufacturer instructions. The methylated DNA fragment was ligated briefly with adaptors. After purification, PCR reaction was done with the adaptor-ligated DNA and the index primer for multiplexing sequencing. Library was purified using magnetic beads to remove all reaction components. The size of the library was assessed by Agilent 2100 bioanalyzer (Agilent Technologies, Amstelveen, The Netherlands). High-throughput sequencing was performed as single-end 75 sequencing using the NextSeq 500 (Illumina, Inc., USA).

#### Data analysis

Sequence reads were trimmed and aligned using Bowtie2 (Langmead and Salzberg, 2012). Bowtie2 indices were either generated from genome assembly sequence or the representative transcript sequences for aligning to the genome and transcriptome. We used MACS (Model-based Analysis of ChIP-Seq) to identify peaks from the alignment file. Gene classification was based on searches done from Medline databases.

### Quantitative real-time PCR (qPCR)

ChIP-ed DNA and RNA samples from the striatum were analyzed by individual qPCR experiments. Total RNA samples from the striatum region were extracted and reverse transcribed via ReverTra Ace™ qPCR RT Master Mix (TOYOBO). For ChIP-ed DNA, ChIP-qPCR reactions for 17 genomic regions around 15 genes were performed with the appropriate primer sets to confirm the ratio of the input DNA between IP-positive and IP-negative samples. For RNA samples, *C4b, Mctp1, Dcaf12l2, Prss45, Rplp* and *GAPDH* were analyzed by RT-qPCR with the appropriate primer sets using cDNA via THUNDERBIRD™ SYBR® qPCR Master Mix (TOYOBO).

### Multi-electrode array

For solutions, standard artificial cerebrospinal fluid (ACSF) was made using: 125 mM NaCl, 2.5 mM KCl, 1.25 mM NaH_2_PO_4_, 1.9 mM MgSO_4_, 20 mM Glucose, 25 mM NaHCO_3_, 2 mM CaCl_2_. Dissection ACSF was made using the standard ACSF without CaCl_2_. For slice preparation and recording, mice were deeply anaesthetized and perfused with ACSF. Brains were dissected out and immediately immersed in ice-cold dissection ACSF. After brains were glued onto the vibratome tray, slices were cut and bubbled with carbogen (95% O_2_ and 5% CO_2_) during slicing. The slices were transferred to carbogen-bubbled warm ACSF (35 °C) and were allowed to recover for at least 1 hr before placement on Axion BioSystems Maestro multielectrode array. Slices were carefully positioned flat on MEA surface for recording. Neuronal activity of firings induced by electrical stimulations were detected on the plate and number of firings from each stimulation current with firing heat-maps were analyzed via software AxIS Navigator™ (2.0.4.).

### Statistical analysis

Student's unpaired t-test was used to compare data from WT group and APP/PS1 for all PA, YMT, 3CT and western blot analysis of MeCP2 expression. One-way ANOVA was used to analyze the effect of APP/PS1 and lentivirus-mediated shMeCP2 groups for comparison of 3 groups in the analysis. Post hoc comparisons were performed using the Bonferroni's multiple comparisons test method after ANOVA analysis. P-values smaller than 0.05 were considered to be significantly different among conditions from the statistical results and all data are presented with box and whiskers plot with all individual data points.

## Supplementary Material

Supplementary figures and table.

## Abbreviations

AD: Alzheimer's disease
APP: amyloid precursor protein
ChIP-seq: chromatin immunoprecipitation sequencing
ICVA: intracerebroventricular stereotaxic injection of Aβ_1-42_ oligomer solution
IGV: integrative genomics viewer
IHC: immunohistochemistry
KEGG: Kyoto encyclopedia of genes and genomes
MEA: multi-electrode array
MeCP2: cerebral methyl-cpg binding protein 2
PA: passive avoidance test
PS1: presenilin 1
YMT: y-maze test
3CT: 3-chamber test
