# Supplementary material for: Dysfunction of striatal MeCP2 is associated with cognitive decline in a mouse model of Alzheimer's disease
Source: Theranostics. 2022 Jan 1;12(3):1404–18. doi: 10.7150/thno.68439 (PMC8771549; doi:10.7150/thno.68439)
Supplement: Supplementary file 1 — Supplementary figures and table. [file thnov12p1404s1.pdf]

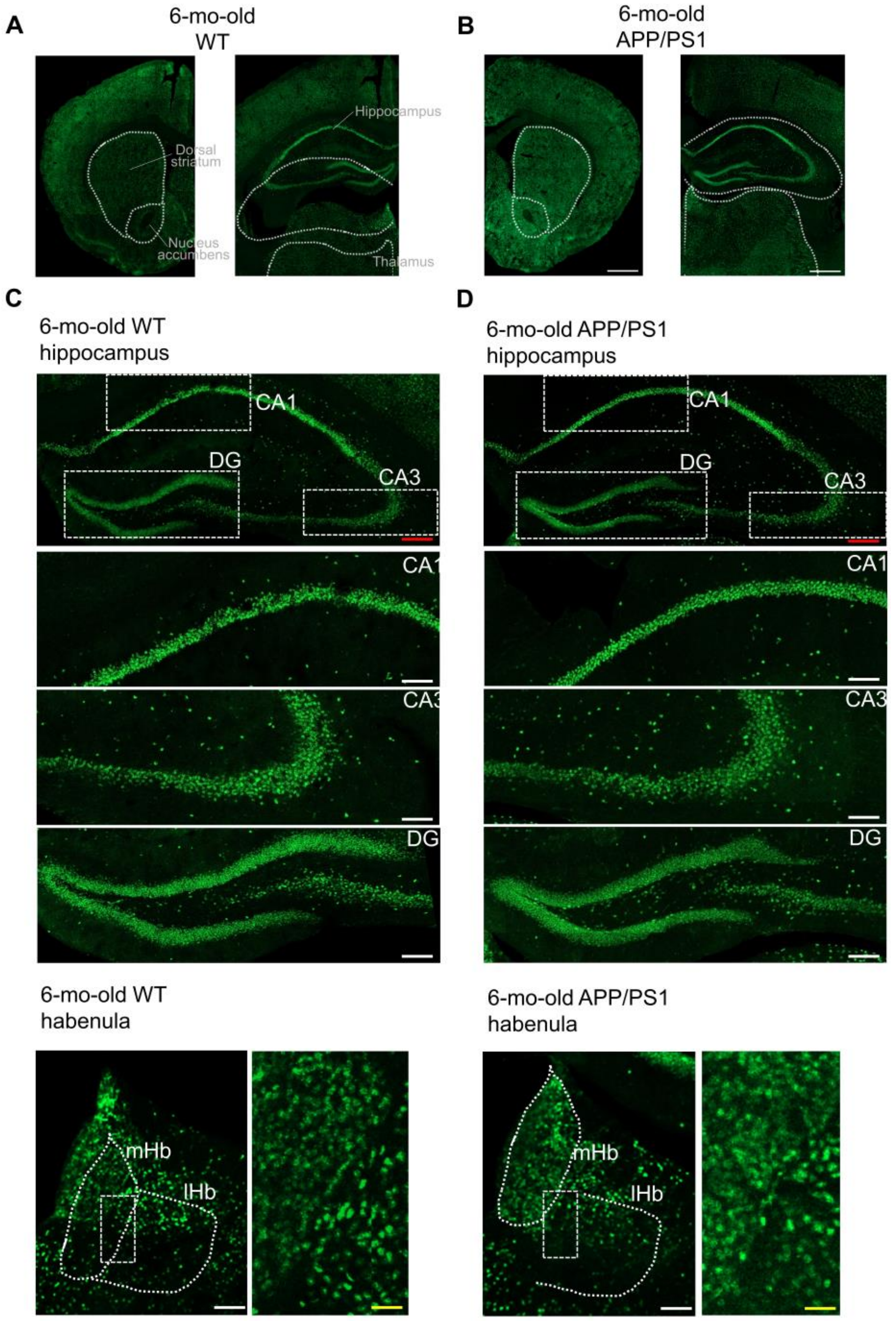

Supplementary Fig. 1 Expression level of MeCP2 in hippocampus and habenula showed no differences. (**A, B**) MeCP2 IHC staining of coronal sections including the striatal region of 6-mo WT and 6-mo APP/PS1 mice. IHC showed increased levels of MeCP2 expression in the dorsal striatum and the nucleus accumbens. Similar levels of MeCP2 signals found in comparing hippocampus and thalamus of WT and APP/PS1 mice. Scale bar: 800 μm **(C, D)** IHC analysis of the MeCP2 expression level in coronal sections showing 3 subregions of the hippocampus; CA1, CA2 and DG. For lower panels, MeCP2 IHC images of medial habenula (mHb) and lateral habenula (lHb) in the epithalamic regions of WT and APP/PS1 mice are shown. Rectangles with dotted line indicate confocal microscopy magnified regions. Red scale bars: 200 μm, white scale bars: 100 μm, yellow scale bars: 40 μm.

A

Group 2 704 gene symbol table

Aadat	Cdh18	E030030I06Rik	H2afb1	Malat1	Olfr1274-ps	Rbms3	Tbc1d2b	Zfp600	A130030D18Rik	Gm8579
Aard	Cdh20	E330013P04Rik	H2al1n	Malt1	Olfr1306	Rbmy	Tbl1xr1	Zfp746	A430085M09Rik	Gm8882
Abca8b	Cdh6	E330016L19Rik	Hdx	Mamld1	Olfr161	Rex2	Tbx20	Zfp791	A530032D15Rik	Gm9758
Abcb11	Cdh9	E330034G19Rik	Hectd2os	Map2k6	Olfr248	Rfc3	Tbx22	Zfp825	A630089N07Rik	Gm9839
Abhd6	Cdk5rap1	Eda2r	Hexb	Map3k7	Olfr502	Rfx3	Tcp10c	Zfp945	A830018L16Rik	
Acacb	Cdk5rap2	Edn1	Hgsnat	Mast4	Olfr609	Ric8b	Tcstv3	Zfp949	AA545190	
Adam21	Cdk8	Eea1	Hic2	Mbnl1	Olfr711	Rnf217	Tdrd3	Zfp981	Gm10033	
Adcyap1	Cdkn1a	Eef2	Hif1a	Mdga2	Olfr806	Robo2	Tenm4	Zfp982	Gm10354	
Adgre5	Celf2	Efna5	Hnf4g	Mefv	Olfr854	Ror2	Tex13c1	Zfp985	Gm10649	
Adgrl2	Cenpw	Ell3	Hnrnpa1l2-ps2	Mfng	Ophn1	Rp1	Tex35	Zfp987	Gm10791	
Adgrl3	Cep295	Elovl5	Hpf1	Mir101c	Osbpl1a	Rplp1	Tex47	Zfp992	Gm1110	
Agbl4	Cept1	Epha6	Hpse2	Mir148a	Oxgr1	Rpp21	Tfap2b	Zmynd11	Gm11217	
Ajap1	Cerk	Epha7	Hrasls	Mir205	P3h2	Rprl1	Tfec	Znhit1	Gm11487	
Aldh9a1	Cfap54	Epm2a	Hs6st3	Mir5127	P4ha2	Rprl2	Them5	Zpld1	Gm12169	
Amot	Cfap69	Erdr1	Hsbp1	Mir5623	Pabpc5	Rps29	Thsd7a	Zrsr1	Gm12887	
Ank1	Chil5	Esp24	Hsd11b1	Mir6236	Pag1	Rsph3a	Tia1	Zswim6	Gm13034	
Ankar	Chl1	Esp38	Hsfy2	Mir6335	Pak3	Rsph3b	Tiam2	Zwint	Gm13212	
Ankle1	Chst11	Esr1	Htra3	Mir6336	Pan3	Satl1	Timm21	1500009L16Rik	Gm13238	
Ano2	Chst2	F2rl3	Ica1	Mir6356	Pcdh15	Scg2	Tle1	1700009N14Rik	Gm13580	
Ano5	Cldn34d	Fam107a	Ifna13	Mir6370	Pcdh18	Schip1	Tle4	1700019E08Rik	Gm13710	
Apbb1ip	Cntn5	Fam171a1	Ifnk	Mir6393	Pcdh7	Sdk2	Tmem114	1700019M22Rik	Gm14296	
App	Cntnap2	Fam172a	Igf2bp3	Mir6965	Pclo	Serpinb9f	Tmem125	1700020D14Rik	Gm14496	
Arhgap21	Cntnap5a	Fam174a	Ighmbp2	Mir704	Pcnx2	Setd4	Tmem18	1700047E10Rik	Gm14525	
Arhgap26	Cntnap5c	Fam213b	Ikzf2	Mir7211	Pde1c	Sgk3	Tmem196	1700049L16Rik	Gm14632	
Arhgef1	Cobl	Fam43a	Il1rapl1	Mir7224	Pepd	Siglech	Tmem267	1700064M15Rik	Gm14851	
Arhgef26	Col11a1	Farp1	Il31ra	Mir8105	Pex2	Skint10	Tmem56	1700110I01Rik	Gm15246	
Arnt	Col19a1	Fbxo33	Incenp	Mkx	Phf21a	Skint6	Tmx3	1700128A07Rik	Gm15679	
Arrdc3	Col4a6	Fbxw4	Inpp4b	Mogat1	Phxr4	Slc15a2	Tmx4	1700129C05Rik	Gm16551	
Asmt	Col6a6	Fbxw7	Inpp5f	Mrgpra4	Pigk	Slc16a14	Tnks	2010315B03Rik	Gm16973	
Atf2	Copz1	Fcrl5	Ipmk	Mrgprd	Pisd	Slc16a9	Trhde	2210408I21Rik	Gm17019	
Atg4c	Cp	Fgf12	Ipw	Mrpl3	Pisd-ps1	Slc22a12	Trim50	2310030A07Rik	Gm17644	
Atp5g1	Cpeb2	Fgf14	Iqcm	Mrto4	Pisd-ps2	Slc24a3	Triqk	2310043M15Rik	Gm17660	
Auts2	Cps1	Fign	Isl1	Msl2	Pitrm1	Slc30a4	Trpm1	2610316D01Rik	Gm19689	
AW209491	Cpxcr1	Filip1l	Itga1	Msl3l2	Pitx2	Slc39a10	Tslp	2700054A10Rik	Gm19782	
Axin2	Cr2	Fmn1	Itga11	Mtpap	Pknox1	Slc6a14	Tspan3	2810429I04Rik	Gm1993	
B230118H07Rik	Creb3l1	Fndc1	Itga9	Mtrr	Pla2g12b	Slitrk1	Twist1	4921504E06Rik	Gm20063	
B230307C23Rik	Crebzf	Focad	Itgad	Muc6	Platr9	Slitrk5	Txndc11	4930428G15Rik	Gm20268	
B3galnt2	Crp	Frmd4a	Itln1	Mug-ps1	Plcb4	Smarca5	Tyrp1	4930433N12Rik	Gm20362	
Bambi	Csf2ra	Frmd4b	Jam3	Mydgf	Plch1	Smarca5-ps	Tyw1	4930448K20Rik	Gm20554	
Batf3	Csk	Frmpd1	Jarid2	Myl12b	Plcl2	Smc5	Ubtfl1	4930459C07Rik	Gm20594	
Bbox1	Csmd3	Frmpd4	Kcnd2	Mylip	Plekha1	Smim10l1	Uchl5	4930467E23Rik	Gm20738	
BC048403	Cyp2b19	Fscb	Kcnh1	Myo7a	Plrg1	Smim13	Ufl1	4930474H20Rik	Gm20740	
Bmpr1b	Cyp2c40	G530011O06Rik	Kcnh5	Myom2	Plxdc2	Smo	Unc5d	4930503E24Rik	Gm20752	
Brinp3	Cyp2c50	G630055G22Rik	Kcnip1	Mzt1	Plxnc1	Snca	Upp2	4930504C09Rik	Gm20755	
Brox	Cyp2c66	Gabrg2	Kcnip4	Nalcn	Podxl	Snord37	Vmn1r2	4930515G16Rik	Gm20826	
Btbd35f12	Cyp2c69	Gad2	Kcnk2	Nampt	Polr3k	Snrpe	Vmn1r238	4930517M08Rik	Gm20939	
Btbd35f27	Cyp4a12b	Galnt5	Kcnmb2	Nbea	Pou4f1	Sos2	Vmn1r60	4930521E06Rik	Gm21083	
Btbd35f28	Cypt12	Galr1	Kcnv1	Ndufb3	Ppfia2	Sox5os3	Vmn1r77	4930529K09Rik	Gm21119	
C130026I21Rik	Cyyr1	Gfra1	Kctd16	Nectin3	Ppp1r9a	Sp110	Vmn2r102	4930543N07Rik	Gm21190	
C230029F24Rik	D030025E07Rik	Ggh	Khdc1b	Nedd4l	Ppp2r2b	Spag17	Vmn2r114	4930556G01Rik	Gm21379	
C330011F03Rik	D030045P18Rik	Gnai1	Klf5	Nek10	Ppp4r2	Speer4c	Vmn2r120	4930558G05Rik	Gm21814	
Cadps	D130009I18Rik	Gnb1l	Klhdc4	Nemf	Ppp6c	Speer4cos	Vmn2r29	4930559C10Rik	Gm29669	
Camsap2	D430019H16Rik	Gng4	Klhl4	Neto1	Prcp	Speer4e	Vmn2r72	4930572O03Rik	Gm32461	
Capn8	D5Ertd579e	Gnpda2	Klrb1b	Nfasc	Prex2	Speer4f1	Vmn2r77	4930578E11Rik	Gm32856	
Car10	Dapk1	Gphn	Kpna1	Nfe2l2	Prkdc	Speer5-ps1	Vmn2r87	4930590L20Rik	Gm32921	
Cartpt	Ddhd1	Gpm6a	Ktn1	Nkain3	Prnd	Speer8-ps1	Vps45	4930595M18Rik	Gm35496	
Catsperb	Ddx18	Gpr12	Lama1	Nkx2-6	Prnp	Sspn	Wasf3	4931408C20Rik	Gm38397	
Cbr3	Dlgap2	Gpr151	Large1	Nobox	Pros1	Ssxb8	Wdfy1	4931420L22Rik	Gm4303	
Ccdc3	Dmd	Gpx7	Lbr	Nol11	Ptchd3	Stag1	Wls	4932411N23Rik	Gm4710	
Ccdc73	Dmrt1i	Gramd3	Lhfpl2	Nova1	Ptger3	Ston1	Wnt16	4933402J10Rik	Gm4850	
Ccdc90b	Dnah6	Greb1	Limd1	Nrxn1	Ptprn2	Stxbp4	Wwox	4933402J15Rik	Gm4981	
Ccng2	Dnah7b	Gria1	Lipo2	Nsfl1c	Ptprt	Stxbp5	Xcr1	4933407K13Rik	Gm5347	
Ccnh	Dock2	Grid2	Litaf	Nt5e	Ptrhd1	Sult2a1	Ybx3	4933413L06Rik	Gm5458	
Ccr1	Dok6	Grin3a	Lmbrd1	Ntrk3	Rab10os	Sympk	Ythdf3	4933422A05Rik	Gm5935	
Cd180	Dpp10	Grp	LOC100861615	Nxph1	Rab39b	Tab2	Zdhhc3	4933427E13Rik	Gm6194	
Cd209a	Dpy19l1	Gsto2	Lrrc4c	Nyap1	Ranbp3l	Tas2r114	Zfp120	6820431F20Rik	Gm6760	
Cd300c	Drd2	Gtf2ird1	Lrrn1	Oasl1	Rap2b	Tas2r115	Zfp369	8430422H06Rik	Gm7102	
Cd44	Drosha	Gtpbp4	Lrrn3	Olfm4	Rarb	Tbc1d14	Zfp458	9330117O12Rik	Gm7134	
Cdh11	Dspp	Gtsf1	Ly6c2	Olfr1189	Rassf9	Tbc1d22a	Zfp534	9530002B09Rik	Gm7827	
Cdh12	Dytn	Gulp1	Mageb18	Olfr1200	Rbfox1	Tbc1d23	Zfp598	9530026F06Rik	Gm7977	

B

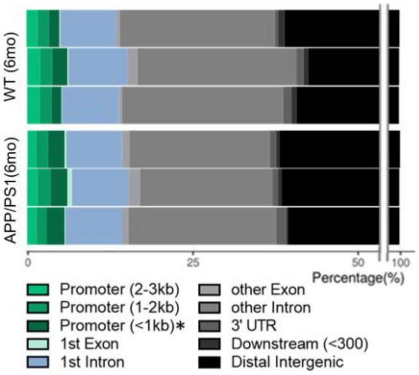

C

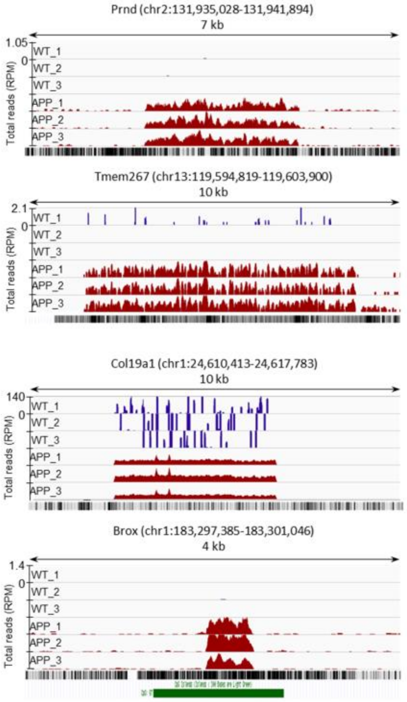

D

Gene ontology analysis for Groups2 704 genes

Category	Term	P-Value	Fold Enrichment
KEGG_PATHWAY	mmu05030:Cocaine addiction	0.039	3.866
KEGG_PATHWAY	mmu00590:Arachidonic acid metabolism	0.002	3.831
KEGG_PATHWAY	mmu04512:ECM-receptor interaction	0.008	3.444
KEGG_PATHWAY	mmu05204:Chemical carcinogenesis	0.010	3.294
KEGG_PATHWAY	mmu04360:Axon guidance	0.020	2.643
GOTERM_BP_DIRECT	GO:0021756~striatum development	0.005	10.969
GOTERM_BP_DIRECT	GO:0035810~positive regulation of urine volume	0.005	10.969
GOTERM_BP_DIRECT	GO:0042129~regulation of T cell proliferation	0.032	10.470
GOTERM_BP_DIRECT	GO:1901379~regulation of potassium ion transmembrane transport	0.006	10.238
GOTERM_BP_DIRECT	GO:0060134~prepulse inhibition	0.006	10.238
GOTERM_MF_DIRECT	GO:0004609~phosphatidylserine decarboxylase activity	0.100	19.256
GOTERM_MF_DIRECT	GO:0004708~MAP kinase kinase activity	0.021	12.837
GOTERM_MF_DIRECT	GO:0004435~phosphatidylinositol phospholipase C activity	0.009	9.062
GOTERM_MF_DIRECT	GO:0030676~Rac guanyl-nucleotide exchange factor activity	0.050	8.253
GOTERM_MF_DIRECT	GO:0004620~phospholipase activity	0.056	7.702
GOTERM_CC_DIRECT	GO:1990761~growth cone lamellipodium	0.077	25.256
GOTERM_CC_DIRECT	GO:0034705~potassium channel complex	0.033	10.332
GOTERM_CC_DIRECT	GO:0060077~inhibitory synapse	0.002	9.020
GOTERM_CC_DIRECT	GO:0030673~axolemma	0.013	7.976
GOTERM_CC_DIRECT	GO:0044291~cell-cell contact zone	0.073	6.685

E

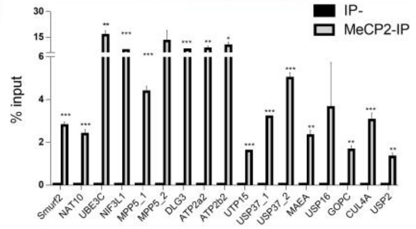

F

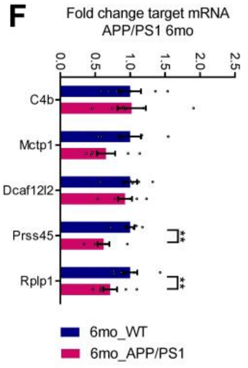

G

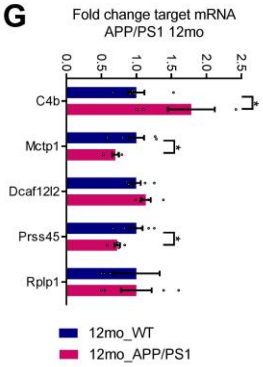

H

Gene	Ref.	Neuronal function
**C4b**	*Mol Psychiatry* (2021)	Protective effect of complement component 4 (C4A, C4B) and HSPA2 to the APOE ε2 in Alzheimer disease
**Mctp1**	*J Neurochem* (2015, 2016)	Calcium sensor for the baseline neurotransmitter release and for the induction and long-term maintenance of presynaptic homeostatic plasticity.
**Dcaf12l2**	-	-
**Prss45**	-	-
**Rplp1**	*PLoS One* (2014) *Mol Brain* (2021)	Ribosomal protein for embryonic development of the nervous system

Supplementary Fig. 2 MeCP2-ChIP-seq analysis revealed various biological pathways which were altered by dysregulated binding of MeCP2. (A) Table of the 704 target genes of which promoter regions were heavily occupied by MeCP2 in the APP/PS1 mice compared with the WT group**. (B)** Divided bar charts of individual samples showing the coverage of MeCP2 bound regions of the WT and APP/PS1 groups (WT n = 3; APP/PS1 n = 3). Colored boxes indicate corresponding binding regions on the bar chart**. (C)** Representative binding pattern images of IGV analysis showing de-novo binding patterns of the accumulated read count peaks of the ChIPed-DNA fragments from selected regions. Green bars indicate the CpG island regions detected in the UCSC genome browser. **(D)** Via DAVID functional annotation bioinformatics analysis, KEGG pathways and GO terms of group2 genes turned out to be involved in multiple biological pathways associated with neurodegenerative diseases. **(E)** ChIP-qPCR analysis of selected target regions using MeCP2-ChIPed genomic DNA. Experimental control of ChIP reaction was labelled as IP- and results were presented in percentage of total input amount (IP- n = 3; MeCP2-IP n = 3; unpaired *t*-test, $^{*}P < 0.05$, $^{**}P < 0.01$, $^{***}P < 0.001$). **(F-G)** RT-qPCR analysis of selected target genes from RNA sample of striatum of 6-month and 12-month old mice (for each WT n = 6; APP/PS1 n = 6; unpaired *t*-test, $^{*}P < 0.05$, $^{**}P < 0.01$). **(H)** Reported function of the selected target genes in relation to AD and other functions in the brain.

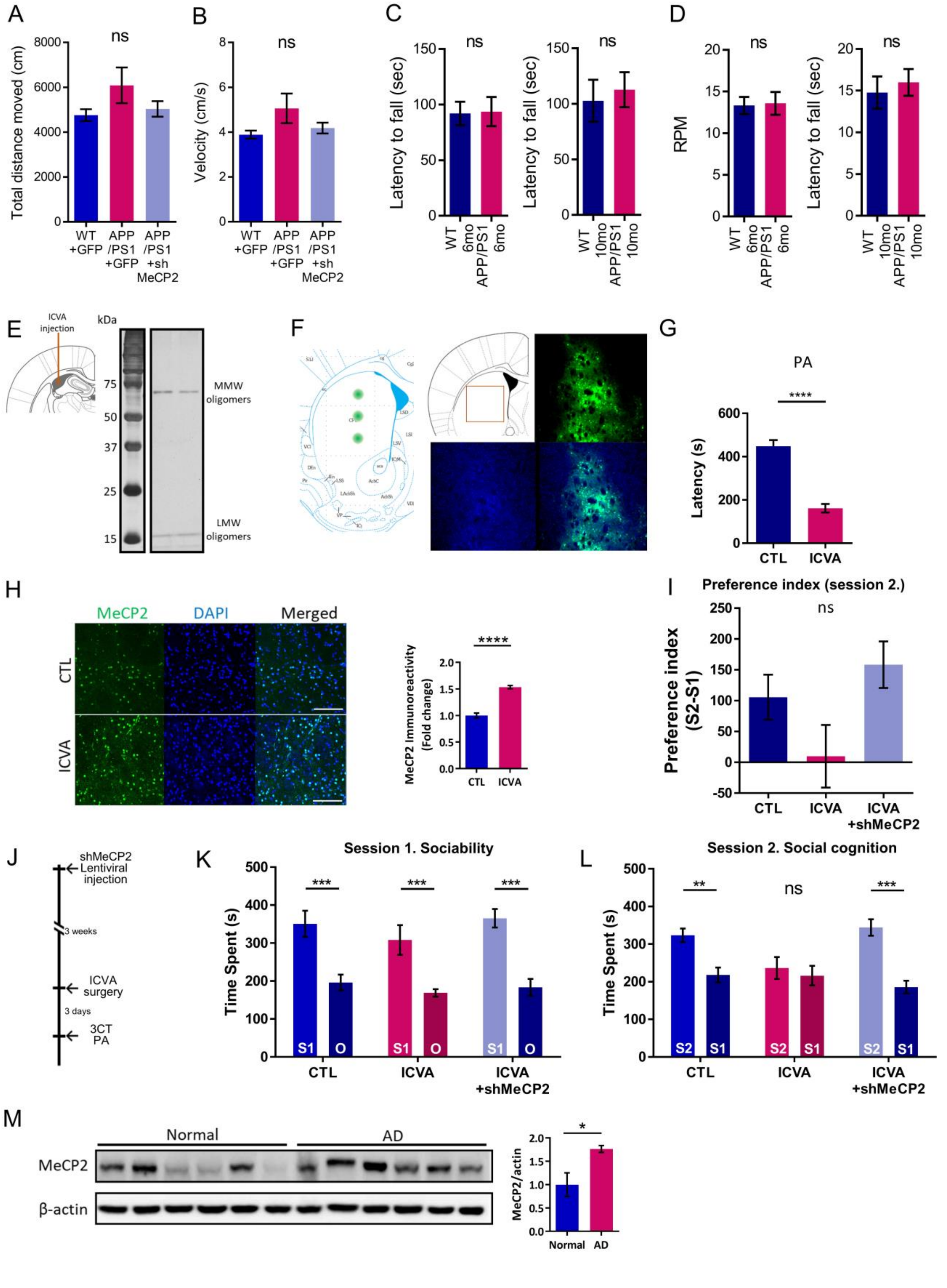
A
ns
Total distance moved (cm)
WT +GFP
APP /PS1 +GFP
APP /PS1 +sh MeCP2
B
ns
Velocity (cm/s)
C
ns
Latency to fall (sec)
WT 6mo
APP/PS1 6mo
WT 10mo
APP/PS1 10mo
D
RPM
E
ICVA injection
kDa
MMW oligomers
LMW oligomers
F
G
PA
Latency (s)
CTL
ICVA
H
MeCP2
DAPI
Merged
MeCP2 Immunoreactivity (Fold change)
I
Preference index (session 2.)
Preference index (S2-S1)
ICVA +shMeCP2
J
shMeCP2 Lentiviral injection
3 weeks
ICVA surgery
3 days
3CT PA
K
Session 1. Sociability
Time Spent (s)
L
Session 2. Social cognition
M
Normal
AD
MeCP2
β-actin
MeCP2/actin

Supplementary Fig. 3 ICVA mouse showed impaired behaviors in the social novelty seeking. (A) Total distance moved during Session 1 and Session 2 of 3CT. (**B)** Velocity of movement during 3CT (for **(A)** and **(B)**, mean ± SEM, WT+GFP n = 6; APP/PS1+GFP n = 3; APP/PS1+shMeCP2 n = 8, ns: not significant). (**C)** Latency to fall in the RTT of APP/PS1 and WT mice. **(D)** RPM to fall from the accelerating rotarod (for **(C)** and **(D)**, mean ± SEM, (6-mo WT n = 9; 6-mo APP/PS1 n = 8; 10-mo WT n = 3; 10-mo APP/PS1 n = 6, ns: not significant). **(E)** Illustration of brain coronal sections representing the target location of ICV injection of $A\beta_{1\text{-}42}$, and silver staining of $A\beta_{1\text{-}42}$ solution prepared for ICV injection showed two bands of MMW (middle molecular weight) oligomers and LMW (low molecular weight) oligomers. (**F)** Virus-injected tissue showed widespread expression of GFP from the injection site throughout the entire striatum. (**G)** PA test of ICVA mice and vehicle control. The time elapsed to escape the dark chamber is shown as latency in seconds (CTL n = 10; ICVA n = 12; unpaired *t*-test, ****$P < 0.0001$). (**H)** MeCP2 IHC (green) and DAPI (blue) staining of the striatum in ICVA mice. Scale bars: 100 μm. Signal intensity of MeCP2 immunoreactivity was quantified and displayed as fold change in MeCP2 immunoreactivity (CTL n = 4; ICVA n = 4; unpaired *t*-test, ****$P < 0.0001$) (**I)** Preference index of the second session of the 3CT, as the difference in total time spent in the S2 chamber as to the S1 chamber for CTRL, ICVA, and ICVA+shMeCP2 mice (CTL n = 4; ICVA n = 6; ICVA+shMeCP2 n = 8, ns: not significant). (**J)** Experimental schedule of behavioral tests after the stereotaxic injection of shMeCP2 virus. Behavioral tests were conducted 3 weeks after the surgery and 3 days after the ICVA treatment. (**K)** Time spent in chambers shown as a measure of sociability in the first session of the 3CT for CTL, ICVA, and ICVA+shMeCP2 groups (CTL n = 4; ICVA n = 6; ICVA+shMeCP2 n = 8; unpaired *t*-test, **$P < 0.01$, ***$P < 0.001$). (**L)** Time spent in chambers as a measure of novelty-seeking in the second session of 3CT for CTL, ICVA, and ICVA+shMeCP2 groups (CTL n = 4; ICVA n = 6; ICVA+shMeCP2 n = 8; unpaired *t*-test, **$P < 0.01$, ***$P < 0.001$). (**M)** Increased MeCP2 levels in the postmortem forebrain regions of AD patients. MeCP2 protein levels in the postmortem forebrain region of the brain from AD patients compared to the brains from healthy controls (Normal). Western blot analysis and the following densitometry calculation (Normal n = 6; AD n = 6; unpaired *t*-test, *$P < 0.05$).

Supplementary Table 1. Information of postmortem brain tissue samples from normal subjects and AD patients.

Number	Diagnosis/Case	Age	Sex	Braak stage
1	Normal	87	Female	I
2	Normal	88	Male	I
3	Normal	86	Male	II
4	Normal	87	Female	II
5	Normal	82	Male	I
6	Normal	82	Male	I
1	AD	85	Male	V
2	AD	87	Female	V
3	AD	82	Male	V
4	AD	80	Female	V
5	AD	83	Male	VI
6	AD	89	Male	IV
